# The Role of Prenatal Vitamin D Deficiency in Early Allergic Rhinitis in Neonates in Greece: Insights from a Cross-Sectional Study at the “Tzaneio” General Hospital

**DOI:** 10.3390/clinpract15050089

**Published:** 2025-04-30

**Authors:** Artemisia Kokkinari, Evangelia Antoniou, Eirini Orovou, Maria Tzitiridou-Chatzopoulou, Maria Dagla, Georgios Iatrakis

**Affiliations:** 1 Department of Midwifery, School of Health & Care Sciences, University of West Attica, 12243 Athens, Greece; lilanton@uniwa.gr (E.A.); mariadagla@uniwa.gr (M.D.); giatrakis@uniwa.gr (G.I.); 2 Department of Midwifery, School of Health & Care Sciences, University of Western Macedonia, 54636 Kozani, Greece; eorovou@uowm.gr (E.O.); mtzitiridou@uowm.gr (M.T.-C.)

**Keywords:** vitamin D deficiency, mothers, neonates, allergic rhinitis

## Abstract

**Background:** The role of vitamin D deficiency (VDD) in both mothers and neonates has been suggested as a possible factor in the development of allergic conditions in early infancy, however limited research has investigated this link in relation to allergic rhinitis (AR). This study investigates whether VDD in the mother–newborn dyad is associated with the onset of AR in neonates within the first three days after birth. The aim is to contribute to the understanding of neonatal allergic outcomes related to vitamin D status, which may inform future preventive strategies. This study investigates the role of vitamin D in the early onset of allergic rhinitis (AR) in neonates, specifically within the first three days of life. Although AR typically develops after years of allergen exposure and is rare in children under two, we aimed to explore its occurrence at this early stage. While no clear link was found between vitamin D and early AR onset, further research is needed to explore vitamin D levels at later ages and over longer time frames to clarify the relationship. **Methods:** A cross-sectional study was conducted between September 2019 and January 2022 in a single hospital. The study involved 248 infants born at ≥37 weeks of gestation and their mothers, who were of Greek nationality. The study included mother–infant pairs who met the inclusion criteria. Chi-square tests were applied to analyze the association between maternal or neonatal VDD and the presence of AR in neonates within the first three days after birth. In addition, multiple regression analysis was used to control other potential factors contributing to AR. **Results:** The results showed an unclear relationship between VDD and the onset of AR in neonates within the first three days of life. Although several factors were analyzed, the effect of VDD on the development of AR remained unclear. **Conclusions:** The findings highlight the lack of clarity regarding the effect of maternal and neonatal VDD on the incidence of AR in the immediate neonatal period. Few studies to date have specifically examined the role of VDD in neonatal AR. Further research with larger sample sizes is needed to verify these associations and to guide potential interventions aimed at reducing allergic outcomes in neonates.

## 1. Introduction

Vitamin D is an essential nutrient that plays a crucial role in the regulation of the immune system [1]. It is well-known for its impact on the physiology of calcium and bone health [1], while research also suggests a potential link between VDD and various allergic conditions. However, some studies suggest that VDD, rather than high levels of vitamin D, may increase the likelihood of allergic sensitization, highlighting the complexity of its immunomodulatory effects [2]. VDD has been increasingly recognized as a potential contributor to allergic diseases. Several studies suggest that vitamin D plays a role in modulating the immune system by influencing both innate and adaptive immune responses [3]. It is believed that VDD may disrupt the balance of immune cell regulation, leading to increased susceptibility to allergic diseases, including allergic rhinitis (AR). Despite this, the exact mechanism by which vitamin D affects allergic conditions remains unclear, and studies examining its role in AR are still limited.

Numerous studies, including the work of Bener et al. (2014) [4]), have indicated that VDD may be associated with an increased prevalence of allergic diseases. In particular, the study by Ehlayel et al. (2011) [5], demonstrated a significant correlation between maternal VDD during pregnancy and a heightened risk of adverse perinatal outcomes, including childhood asthma, AR, and respiratory wheezing in offspring. Wegienka et al. (2015) [6], reported that elevated prenatal and cord blood levels of 25(OH)D were generally linked to a lower incidence of allergic conditions, including eczema and sensitization to airborne allergens. This relationship appeared to be more pronounced among white children, whereas it was less evident in black children. Moreover, their findings indicated that 25(OH)D levels were inversely correlated with sensitization to airborne allergens exclusively in black children. A systematic review by Tareke et al. (2020) [7], highlighted a growing body of evidence suggesting an association between VDD and the increased risk of AR, particularly in early childhood. However, the review also indicated that, at present, there is insufficient evidence to support the promotion of vitamin D supplementation during pregnancy specifically for respiratory health in childhood. While the potential benefits of vitamin D for immune function are well-documented, there is a lack of compelling data to recommend routine supplementation in pregnant women for the prevention of allergic respiratory diseases in their children.

AR is a common allergic disorder, primarily mediated by immunoglobulin E (IgE) and triggered by airborne allergens [8]. It manifests clinically through symptoms such as sneezing, nasal congestion, nasal itching, and rhinorrhea [9]. Allergic rhinitis typically requires prolonged exposure to allergens before clinical symptoms emerge. Studies indicate that at least two seasons of pollen exposure are usually necessary before children develop pollen-related allergic reactions, making AR uncommon in children under two years of age. Therefore, if a very young child exhibits persistent nasal symptoms, alternative diagnoses should be considered [10]. The prevalence of AR has been rising worldwide, significantly impacting quality of life, particularly among children. Given its increasing incidence, understanding the underlying factors contributing to its development is crucial for both prevention and treatment strategies. Notably, research has indicated that the relationship between vitamin D levels and AR may be influenced by various factors, including race, age, and sex, suggesting a complex interplay between genetic predisposition and environmental influences [11]. Moreover, substantial evidence highlights that maternal allergic history, reflecting genetic susceptibility, is a strong predictor of allergic disease development in offspring [12,13]. In a prospective cohort study, Bunyavanich et al. (2016) [14], examined 1248 mother–child pairs during the prenatal period and found that for every 100 IU/day increase in dietary vitamin D intake during the first and last trimesters of pregnancy, the likelihood of school-aged children developing AR was reduced by 21% and 20%, respectively.

Nevertheless, some studies have failed to establish a link between vitamin D and AR during the perinatal period. For example, Baiz et al. (2014) [15] conducted a study measuring umbilical cord blood 25(OH)D levels in 239 neonates. Using a questionnaire based on the International Study of Asthma and Allergies in Childhood (ISAAC), they tracked these children until the age of five. Their findings did not support an association between umbilical cord blood 25(OH)D levels and the development of asthma or AR at five years of age in individuals of European descent. These results highlight the need for further large-scale studies to clarify the potential relationship between prenatal vitamin D levels and the risk of AR in neonates.

To date, no systematic review (SR) has specifically evaluated the effectiveness of vitamin D supplementation in neonates with AR. The systematic review by Li et al. (2022) [16] included four randomized controlled trials (RCTs) assessing vitamin D supplementation in children with AR. The findings suggested that vitamin D supplementation significantly alleviated symptoms in affected patients. However, some of these studies had methodological limitations, such as the lack of blinding of participants and researchers, as well as inadequate handling of missing data. Additionally, the review was unable to conduct a quantitative meta-analysis due to heterogeneity in patient outcomes across studies.

Therefore, large-scale, well-designed RCTs are required to validate these findings and determine the optimal vitamin D regimen for neonates in the first three days of life diagnosed with AR. Moreover, further research is necessary to investigate whether an optimal vitamin D supplementation strategy exists for pregnant women to prevent AR in their newborns.

This study aims to explore the potential association between VDD in both mothers and newborns and the development of AR during the first three days of life in the maternity ward. By investigating this relationship, we hope to contribute to a better understanding of the early life factors influencing allergic diseases and highlight the need for further research into the potential preventive role of vitamin D in managing AR. Although the association between vitamin D and AR has been studied in various populations, very few studies have focused on the mother–newborn dyad and the early neonatal period. This is a critical gap in research, as the first days of life may offer unique insights into the immune system’s development and its susceptibility to allergic diseases later in childhood. Given the global rise in AR and the increasing recognition of vitamin D’s role in immune function, understanding how VDD during early infancy influences the development of allergic conditions is of significant clinical importance.

## 2. Materials and Methods

The participants in this study were all pregnant Greek women who came to deliver at Tzaneio General Hospital in Piraeus, as well as those who had lived in Greece for more than a decade. This latter group was included because it was assumed that their prolonged exposure to the Mediterranean climate would contribute to an adequate vitamin D status. The study received approval from the hospital’s scientific committee. Informed consent was obtained from the participants, and both the mothers and their spouses provided consent for the participation of their underage children in the research. To minimize potential bias, the study included all pregnant women attending the hospital, with the exclusion of non-Greek nationals and those under 37 weeks of gestation. Additionally, pregnant women who were known to be taking medications that could interfere with vitamin D metabolism, such as anticonvulsants (e.g., phenytoin, carbamazepine), glucocorticoids, or other immunosuppressive drugs, were excluded. Other exclusions included women with conditions that are known to affect vitamin D metabolism or absorption, such as chronic liver disease (e.g., cirrhosis), kidney disease (e.g., renal failure), gastrointestinal disorders (e.g., celiac disease, Crohn’s disease), obesity, and other conditions that impair vitamin D synthesis, activation, or absorption. The aim of this study was to investigate the association between maternal and neonatal VDD and the development of AR in newborns, particularly during the first three days of life. Additionally, the study aimed to explore the impact of early vitamin D supplementation during pregnancy on respiratory health outcomes in neonates.

Detailed personal questionnaires were administered to collect information on the characteristics of both the mothers and their newborns, with additional data obtained from the medical records. The vitamin D levels (25(OH)D) of the mothers were measured once, at the end of their pregnancy, during the routine laboratory tests conducted upon admission to the hospital. Similarly, the vitamin D levels (25(OH)D) of the newborns were assessed once at birth, using a sample taken from the umbilical cord at the time of delivery. Maternal and neonatal 25(OH)D concentrations were categorized as sufficient for levels > 30 ng/mL and insufficient for levels < 30 ng/mL. These thresholds were used to assess potential associations between vitamin D status and allergic rhinitis in newborns. The blood samples, along with other necessary measurements, were collected by the attending midwife during the birth to ensure the accuracy and consistency of the results. The presence or absence of AR in the newborns was assessed by pediatricians during their initial postnatal evaluation, based on clinical examination and observations, and was monitored throughout their stay in the hospital.

The study was designed to explore the prevalence of AR in newborns and its potential association with maternal and neonatal vitamin D levels during the first three days of life in the maternity ward. The goal was to assess whether there is a correlation between VDD and the development of AR. Additionally, the study aimed to evaluate the factors that may influence this outcome, with a particular focus on the potential role of early vitamin D supplementation during pregnancy. If any significant associations are found, it would suggest that further clinical trials are needed to refine recommendations for vitamin D supplementation during pregnancy and early postnatal care. Furthermore, such findings could highlight the need for healthcare providers to consider these factors when developing personalized care strategies for newborns, particularly those at risk for respiratory issues, to prevent the onset of AR and other respiratory conditions.

Data analysis was carried out using IBM SPSS Statistics, version 26. Descriptive statistics were applied to summarize the demographic and clinical characteristics of the mother–newborn pairs. To examine the relationship between maternal and neonatal vitamin D levels and the development of AR in the newborns, Chi-square tests were conducted to assess associations between VDD and the presence of AR. To examine the potential association between maternal and neonatal 25(OH)D levels and the presence of allergic rhinitis, two separate exposure–outcome analyses were conducted using the Chi-square test. The first analysis assessed the relationship between maternal 25(OH)D levels and allergic rhinitis in newborns, while the second examined whether neonatal 25(OH)D levels were associated with allergic rhinitis. These analyses aimed to determine whether maternal or neonatal vitamin D status played a role in the development of allergic rhinitis during the early postnatal period. Logistic regression analysis was performed to evaluate the effect of various maternal and neonatal factors on the likelihood of AR in newborns. Multiple regression analysis was also utilized to assess the combined influence of other potential factors contributing to the development of respiratory conditions.

Factors that were investigated for their potential impact on the development of AR in newborns included maternal characteristics such as age (18–35 years, 36–42 years, >42 years), smoking status (smokers, non-smokers, or quit during pregnancy), and pre-existing maternal health conditions such as asthma or atopy. Additionally, the maternal vitamin D levels during pregnancy were assessed, classifying them as deficient or sufficient, and any medication use during pregnancy, including corticosteroids and antibiotics, was recorded. The intake or non-intake of vitamin D supplements during pregnancy was also evaluated as a factor that could influence the development of AR in the newborn. Neonatal factors, such as gender (male or female), birth weight (<2500 g or ≥2500 g), mode of delivery (vaginal or cesarean section), and the presence of respiratory issues in the neonatal period, were also evaluated. Furthermore, maternal history of allergies was considered as a potential influencing factor and categorized into two groups: mothers who had experienced at least one episode of allergies (“yes”) and those who had never experienced any allergic episodes (“no”). Although the maternal diet, including whether the infant was exclusively breastfed, not exclusively breastfed, or only formula-fed, was assessed for its potential role, the analysis of food allergies in maternal history was not part of this study. In this study, we focused specifically on clinically diagnosed allergies (not food allergies) such as respiratory allergies (e.g., asthma, rhinitis), rather than maternal food allergies. Future research could explore the potential impact of maternal food allergies on neonatal allergic conditions. It should be noted that the term ‘allergies’ used in this context specifically refers to clinically diagnosed allergic conditions. Although the term ‘atopy’ was not explicitly used, it is understood that a maternal history of allergies may be associated with atopic conditions. However, in this study, we focused specifically on clinically diagnosed allergies rather than a broader definition of atopy. Paternal allergy history was not included in the study design, and therefore, no data were available on this factor. Future research could benefit from incorporating paternal allergy history to explore its potential role in the development of allergic diseases in neonates. Additionally, the measurement of total serum IgE levels in both the mother and neonate could provide an objective measure of allergic predisposition and may offer valuable insight into the mechanisms of allergic rhinitis development. While this was not part of the current study, it is a potential avenue for future research. Maternal diet, including whether the infant was exclusively breastfed, not exclusively breastfed, or only fed formula, was also assessed for its potential role. Lastly, the season of birth was considered, with newborns categorized into two seasonal groups: Group A (born from April to mid-October) and Group B (born from mid-October to the end of March). These factors were selected based on the existing literature, suggesting that they could influence the risk of developing AR in early life. The relationship between maternal and neonatal factors and the occurrence of AR was explored, with a particular focus on the role of maternal vitamin D levels in neonatal immune development.

Τhe dependent variable was the presence or absence of AR in the newborns, while the independent variables included all maternal and neonatal factors previously identified as potential influencers of AR. Data on maternal characteristics such as vitamin D supplementation during pregnancy, smoking habits, pre-existing conditions (e.g., asthma, atopy), and socio-economic status were collected through personal questionnaires. Neonatal factors, including birth weight, gender, mode of delivery, and early respiratory health, were also considered. Each analysis aimed to predict the likelihood of each independent variable influencing the development of AR. The *p*-value was used to assess the statistical significance of each independent variable, with *p*-values (*p*) ≤ 0.05 being considered statistically significant.

## 3. Results

This study included a sample of 248 neonates born to Greek mothers. Pediatricians recorded cases of AR or rhinitis symptoms in neonatal medical history, identifying a total of 33 neonates with documented symptoms within the first three days of life.

A significant association was found between maternal VDD and the occurrence of neonatal AR. Specifically, among the 196 mothers with VDD (defined as serum 25(OH)D < 30 ng/mL), 33 neonates (17%) exhibited AR symptoms, while 163 (83%) did not. In contrast, none of the neonates born to the 52 mothers with sufficient vitamin D levels developed AR.

This difference was found to be statistically significant (*p* = 0.023), suggesting that neonates born to vitamin D-deficient mothers may have an increased risk of developing AR in the early neonatal period.

Examining neonatal vitamin D levels at birth, which were obtained through umbilical cord blood sampling, we further analyzed the relationship between neonatal VDD and AR incidence. Among the 248 neonates, only 10 had sufficient vitamin D levels (≥30 ng/mL), and none of them developed AR. In contrast, 205 neonates were classified as vitamin D-deficient (<30 ng/mL), of whom 33 developed AR. However, the association between neonatal VDD and AR was not statistically significant (*p* = 0.205), as the *p*-value exceeded the 0.05 threshold for statistical significance.

Although none of the neonates with sufficient vitamin D levels exhibited AR—consistent with the findings on maternal vitamin D sufficiency—the lack of statistical significance in the neonatal analysis suggests that other factors may influence the early development of AR.

From the analysis of multiple logistic regression (Table 1), which was conducted to assess the statistical significance of additional factors potentially influencing the onset of allergic rhinitis in neonates, it was found that maternal characteristics did not show a significant association with neonatal allergic rhinitis. Specifically, maternal age (*p* = 0.757), smoking status (*p* = 0.49), and mode of delivery (*p* = 0.182) were not significantly associated with the development of allergic rhinitis in the neonates. Similarly, when examining neonatal characteristics, no significant relationship was observed with the child’s gender (*p* = 0.345) or birth weight (*p* = 0.471).

Additionally, the analysis revealed that neonatal nutrition, specifically whether the infant was breastfed or formula-fed, showed a significant association with the occurrence of allergic rhinitis. This was confirmed by a *p*-value of 0.024. Moreover, maternal allergic history was found to be significantly associated with the development of allergic rhinitis in neonates, with a *p*-value of 0.022, suggesting a potential link between maternal allergic conditions and the neonatal outcome. In contrast, no significant association was observed between the seasonal birth group and the development of allergic rhinitis (*p* = 0.354), indicating that birth seasonality may not be a major contributing factor to the onset of allergic rhinitis in this cohort.

## 4. Discussion

Although AR is typically associated with prolonged exposure to allergens over the course of several years, emerging evidence suggests that the development of allergic conditions, such as AR, may be influenced by prenatal factors, including VDD. This study focused on the onset of AR in neonates within the first three days of life, which is quite early compared to the typical pattern of allergen exposure. The results from this study contribute to the growing body of evidence suggesting that early life factors, including maternal and neonatal VDD, may play a role in the development of allergic diseases. However, it is important to recognize that the manifestation of AR in such early stages is uncommon. As such, the findings of this study, while valuable, should be interpreted with caution.

Despite analyzing several potential factors (such as age, gender, maternal smoking, prenatal care, and other possible risk factors), no statistically significant associations were found between these factors and the development of allergic rhinitis in neonates. This suggests that maternal VDD is the primary factor that appears to influence the development of allergic rhinitis during the neonatal period. However, it is important to recognize the limitations of this study, particularly the challenge of investigating AR in neonates during the early days of life. AR typically requires prolonged exposure to allergens, often spanning at least two seasons of pollen exposure. Given that the neonatal period, within the first three days of life, may not provide sufficient time or exposure for AR to manifest, the timing of our study may have been too early to observe a definitive relationship between neonatal VDD and AR. This limitation emphasizes the complexity of allergic disease development, which likely involves an extended period of immune system maturation and allergen exposure.

Vitamin D is known to play a crucial role in immune system regulation, influencing both innate and adaptive immunity. Prenatal and early postnatal VDD has been linked to immune dysregulation, potentially increasing susceptibility to allergic diseases. Specifically, vitamin D modulates the balance between Th1 and Th2 immune responses, with its deficiency favoring a Th2-skewed immune profile. This imbalance leads to increased IgE production and cytokines such as IL-4 and IL-13, which are involved in allergic inflammation. Additionally, vitamin D enhances the function of regulatory T cells (Tregs), which help maintain immune tolerance. A deficiency in vitamin D may impair Treg function, further increasing the risk of allergic diseases, including AR [17].

Our study found a statistically significant association between maternal VDD and the presence of AR in neonates, which suggests that maternal vitamin D status may play a more significant role in the early onset of AR in neonates than neonatal VDD. This is in line with previous studies that have suggested that maternal health factors can significantly influence neonatal outcomes, including the development of allergic conditions [15]. These studies highlight the importance of prenatal factors, such as maternal vitamin D status, in shaping the immune development of the neonate and its potential susceptibility to allergic diseases.

Interestingly, our findings suggest that maternal history of allergies may also influence the development of allergic rhinitis in neonates. This observation is supported by substantial evidence indicating a strong genetic component in the development of allergic diseases [12,13]. Maternal allergic history may reflect a heritable predisposition to atopy, with several studies identifying polymorphisms in immune-related genes such as IL-4, IL-13, and the filaggrin gene (FLG) that are linked to an increased risk of allergic rhinitis. Genetic predisposition, combined with early environmental exposures, shapes the neonatal immune profile and may increase susceptibility to allergic sensitization [12,13]. Additionally, the type of infant nutrition, specifically breastfeeding, appears to be associated with a reduced risk of allergic rhinitis in the early neonatal period. These findings emphasize the importance of maternal health and infant nutrition in the early prevention of allergic conditions. Specifically, maternal allergic history (*p* = 0.022) and infant nutrition, particularly exclusive breastfeeding (*p* = 0.024), were found to have a significant impact on the onset of allergic rhinitis in neonates. The data suggest that these factors could be integral to understanding the early development of allergic diseases in neonates, alongside maternal vitamin D status.

On the other hand, neonatal VDD did not show a statistically significant association with AR during the first three days of life. Although none of the neonates with sufficient vitamin D levels exhibited AR—consistent with the findings on maternal vitamin D sufficiency—the lack of statistical significance in the neonatal analysis suggests that other factors may influence the early development of AR. This discrepancy may be attributed to several factors, such as the timing of vitamin D exposure and the complex nature of immune system development in neonates, which may require a longer period of exposure to allergens or other environmental factors for the onset of AR to occur. Furthermore, the small sample size of neonates with AR in this study may have contributed to the lack of significant findings for neonatal VDD.

Another important limitation of this study is the relatively small sample size (248 neonates). Additionally, the absence of long-term follow-up of neonates, at least 6 to 12 months, to ensure that the diagnosis of AR is supported in these cases is an important limitation. This follow-up period would allow for the confirmation of AR diagnosis and better understanding of its progression over time. While our findings suggest a potential association between maternal VDD and neonatal allergic rhinitis, larger studies with a broader population are needed to confirm this association and enhance the generalizability of the results. One limitation of our study is the exclusion of paternal allergy history as a factor, as it was not included in the data collection process. Future research could benefit from incorporating paternal allergy history to explore its potential role in the development of allergic diseases in neonates. Larger studies with more neonates diagnosed with AR may provide more definitive answers regarding the role of neonatal vitamin D in the development of allergic conditions.

These findings indicate that the timing and source of vitamin D exposure, especially maternal VDD, may be an important factor to consider in future research investigating the onset of AR in neonates. Moreover, the findings of our previous study Kokkinari A. et al., (2024) [18] support the idea that maternal VDD significantly affects neonatal vitamin D levels. Specifically, our study demonstrated a strong correlation between maternal and neonatal vitamin D concentrations, with neonatal levels being consistently lower than maternal levels, regardless of vitamin D supplementation during pregnancy. These findings underscore the importance of monitoring and addressing maternal VDD as a critical factor in neonatal immune development and the early onset of allergic diseases.

In light of these findings, it is clear that further research is needed to clarify the role of vitamin D in the development of allergic conditions in neonates. Future studies should focus on larger, more diverse populations to examine the impact of maternal and neonatal vitamin D levels on the development of AR. Moreover, other potential factors that could influence neonatal AR, such as genetic predisposition, exposure to environmental allergens, and maternal health during pregnancy, should also be considered. This would contribute to a more comprehensive understanding of the early origins of allergic diseases and inform more effective preventative strategies.

## 5. Conclusions

This study suggests that maternal VDD may play a significant role in the early onset of allergic rhinitis (AR) in neonates, particularly within the first three days of life. While neonatal VDD did not show a statistically significant association with AR, maternal vitamin D status appears to influence neonatal immune development. Maternal allergic history and infant nutrition, particularly exclusive breastfeeding, also seem to have an impact on the early development of AR. The influence of maternal allergic history may partly be explained by inherited genetic susceptibility to allergic disease. Studies have shown that maternal atopy is strongly associated with increased risk of allergic outcomes in offspring, due to both genetic inheritance and shared intrauterine environment.

However, the small sample size and the early timing of the investigation limit the ability to draw definitive conclusions. Additionally, the absence of long-term follow-up of neonates, at least 6 to 12 months, to ensure that the diagnosis of allergic rhinitis is supported in these cases is an important limitation. This follow-up period would allow for the confirmation of AR diagnosis and better understanding of its progression over time.

Further studies with larger populations, longer follow-up periods, and a focus on factors such as genetic predisposition and environmental exposures are needed to clarify the relationship between maternal and neonatal VDD and the development of AR. This would contribute to a more comprehensive understanding of the complex origins of allergic diseases in neonates.

It is important to acknowledge that this was a convenient cross-sectional study, with data collected during a very limited period confined to the neonates’ hospitalization alongside their mothers. Due to the short-term follow-up, the study design restricts the capacity to observe longer-term outcomes and limits the generalizability of the findings. If a true association between maternal VDD and neonatal AR exists, the magnitude of this relationship is unlikely to be substantial based on the current data. Including longer follow-up periods in future research would be essential to better assess the potential impact.

## Figures and Tables

**Table 1:** Maternal and Neonatal Risk Factors Associated with Allergic Rhinitis (AR) in Neonates.

	Allergic Rhinitis (AR)			*p*-Value
**Maternal_Age**			**Total**	0.757
	**AR Presence**	**AR Absence**		
18–35	28	163	191	
35–42	5	45	50	
>42	0	7	7	
Total	33	215	248	
**Vitamin D Supplementation During Pregnancy**				
	**AR Presence**	**AR Absence**	Total	0.325
Received	7	72	79	
Not Received	26	143	169	
Total	33	215	248	
**Type of delivery**				
	**AR Presence**	**AR Absence**	Total	0.183
**Vaginal birth**	6	80	86	
**Cesarean section**	27	135	162	
**Total**	33	215	248	
**Birth weight**				
	**AR Presence**	**AR Absence**	Total	0.471
**≥25** **00 g**	1	25	26	
**<2500 g**	32	190	222	
**Total**	33	215	248	
**Infant’s gender**				
	**AR Presence**	**AR Absence**	Total	0.345
**Male**	23	114	137	
**Female**	10	101	111	
**Total**	33	215	248	
**Newborn Nutrition**				
	**AR Presence**	**AR Absence**	Total	
**Breastfeeding**	1	68	69	
**Breastfeeding and Formula**	20	133	153	0.024
**Formula only**	12	14	26	
Total	33	215	248	
**Season Birth Group**				
	**AR Presence**	**AR Absence**	Total	
Group A	9	99	108	0.354
Group B	24	116	140	
Total	33	215	248	
**Maternal Allergy History**				
	**AR Presence**	**AR Absence**	Total	
Yes	25	17	42	0.022
No	8	198	206	
Total	33	215	248	
**Smoking**			Total	
	**AR Presence**	**AR Absence**		0.49
**Smokers**	9	92	101	
**Non-smokers**	19	78	97	
**Quitters**	5	45	50	
Total	33	215	248	

## Data Availability

The raw data supporting the conclusions of this article will be made available by the authors on request.
